# A luminescence-based method to assess antigen presentation and antigen-specific T cell responses for *in vitro* screening of immunomodulatory checkpoints and therapeutics

**DOI:** 10.3389/fimmu.2023.1233113

**Published:** 2023-07-25

**Authors:** Jimena Álvarez Freile, Yuzhu Qi, Lisa Jacob, Maria Franceskin Lobo, Harm Jan Lourens, Gerwin Huls, Edwin Bremer

**Affiliations:** Department of Hematology, University of Groningen, University Medical Center Groningen, Groningen, Netherlands

**Keywords:** *luciferase*, antigen-specific, T cells, antigen presentation, MHC-I, immune checkpoints

## Abstract

Investigations into the strength of antigen-specific responses *in vitro* is becoming increasingly relevant for decision making in early-phase research of novel immunotherapeutic approaches, including adoptive cell but also immune checkpoint inhibitor (ICI)-based therapies. In the latter, antigen-specific rapid and high throughput tools to investigate MHC/antigen-specific T cell receptor (TCR) activation haven’t been implemented yet. Here, we present a simple and rapid luminescence-based approach using the human papillomavirus 16 (HPV16) E7_11-20_ peptide as model antigen and E7-TCR transgenic Jurkat.NFAT-*luciferase* reporter cells. Upon E7 peptide pulsing of HLA-A2^+^ cell lines and macrophages, an effector to target ratio dependent increase in luminescence compared to non-pulsed cells was observed after co-incubation with E7-TCR expressing Jurkat, but not with parental cells. Analogous experiments with cells expressing full-length HPV16 identified that E7-specific activation of Jurkat cells enabled detection of endogenous antigen processing and MHC-I presentation. As proof of concept, overexpression of established checkpoints/inhibitory molecules (e.g., PD-L1 or HLA-G) significantly reduced the E7-specific TCR-induced luminescence, an effect that could be restored after treatment with corresponding targeting antagonistic antibodies. Altogether, the luminescence-based method described here represents an alternative approach for the rapid evaluation of MHC-dependent antigen-specific T cell responses *in vitro*. It can be used as a rapid tool to evaluate the impact of the immunosuppressive tumor microenvironment or novel ICI in triggering effective T cell responses, as well as speeding up the development of novel therapeutics within the immune-oncology field.

## 1 Introduction

The study of antigen-specific T cell responses *in vitro* typically require the isolation and expansion of primary T cells (1–4). However, one of the main drawbacks of these approaches is the limited lifespan of the resulting cells. Together with the cost- and labor- intensive protocols and inter-donor variability (5, 6), this has motivated the development of immortalized systems to assess antigen specific TCR-mediated activation in a simpler manner *in vitro.* In this sense, both luminescence and fluorescence-based assays have been reported, offering simplicity and speed. For instance, fluorescent-based reporter systems have been described to evaluate transcription factors of relevance in T cell activation (AP-1, NFAT, NF-kB) upon specific interaction with tumor and viral antigens (7–9), driving the expression of the fluorescent proteins such as CFP, eGFP or mCherry. Based on the Jurkat leukemic T-cell line, high throughput evaluation of TCR function, with TCR functional avidity correlating to primary T cells has been described (10). In contrast to fluorescence, which relies on photons as energy source, bioluminescence is derived from naturally catalyzed processes and can have increased assay sensitivities compared to fluorescence assays (11). In particular, *luciferase* (luc) activity under the NFAT transcription factor promoter (NFAT-luc) has been incorporated into different Jurkat systems to functionally validate and/or identify novel antigen-specific TCRs (12–14). For instance, a Jurkat NFAT reporter cell line expressing melanoma-specific TCRs was reported to trigger antigen-specific luminescence, allowing for the identification of novel tumor antigens and functional evaluation of cloned TCRs, a crucial task in the development of TCR-based therapies.

Although previous studies have mostly focused on TCR-related research, the applicability of this luminescence-based method can be expanded beyond. In particular, despite other fluorescence and luminescence-based Jurkat reporter assays being widely used for the evaluation of checkpoints in a non-specific TCR manner [i.e., an anti-CD3 scFv-based systems (15–17)], the use of an antigen-specific or MHC-dependent reporter system is preferred when specific impact on antigen presentation is to be evaluated. For instance, since the efficacy of immune checkpoint inhibitors (ICIs)-based therapies relies on re-activation of T cells, alterations in antigen processing and presentation can result in impaired antitumor responses and therapy resistance, as recently described for PD-1/PD-L1 treatment (18–20). Accordingly, it is becoming increasingly evident that alterations in antigen presentation are often found in tumors and a key resistance mechanism for ICI-based therapies (21–24). Indeed, not only cell-surface expression of MHC is downregulated (18, 19, 21, 25–27), but also the repertoire of antigenic peptides that is presented to T cells (immunopeptidome) is altered (28–30). Therefore, the study of antigen-specific TCR-mediated responses in the context of the immunosuppressive tumor microenvironment (TME) and checkpoint inhibition, within both adaptive and innate immunity, may be of great importance to optimize and develop novel immunotherapeutic approaches. Antigen-specific Jurkat reporter cells could be implemented in the screening of the effects of therapeutics [as previously described (14)], but also to assess the effect of specific molecules overexpressed within the TME on antigen-specific TCR recognition. Overall, the generation of an antigen-specific Jurkat reporter system expressing an antigen-specific TCR against an easily available cognate antigen is a potentially useful “universal” tool to monitor immunomodulation in an antigen-specific manner in several areas of the immune-oncology field.

Here, we describe the validation of an antigen-specific Jurkat reporter system using genetically engineered Jurkat.NFAT-luc cells expressing a high-affinity TCR towards a commercially available human papillomavirus 16 (HPV16) E7 peptide (E7-TCR). Luminescence was specifically produced only upon antigen-specific activation of the E7-TCR transgenic Jurkat.NFAT-luc cells with cognate antigen. To validate the hypothesis that these antigen-specific systems are useful tools to assess immunomodulation in the context of antigen-specific T cells responses, well-established inhibitory receptors (i.e., HLA-G, PD-L1) and targeted therapeutics (i.e., anti-HLA-G, anti-PD-L1 antibody Atezolizumab) were evaluated. The method developed here proved to be as specific as traditional functional assays using primary T cells. Moreover, this system enabled the evaluation of the impact of macrophage polarity on T cell responses. Further, the assay had equal sensitivity to non-specific ubiquitous TCR activation methods (i.e., an anti-CD3 scFv-based systems (Ref)). The sensitivity of the method could be increased by replacing *Firefly* with *Gaussia luciferase*, which might be important for further applications, such as in the context of antigen cross-presentation upon phagocytosis of cancer cells. Overall, the luminescence-based method presented here can be used as a rapid tool to evaluate the impact of the immunosuppressive TME to optimize the development of novel immunotherapeutic approaches.

## 2 Materials, equipment, and methods

### 2.1 Reagents

HPV16-E7_11-20_ (E7_11-20_) peptide (YMLDLQPETT) was purchased from Peptides & Elephants (Berlin, Germany). Anti-CD25-APC (Clone MEM-181), Granulocyte-macrophage colony-stimulating factor (GM-CSF), macrophage-colony-stimulating factor (M-CSF), interferon-γ (IFN-γ), interleukin 10 (IL-10), TNF-α and IFN-γ ELISA kits were purchased from Immunotools (Friesoythe, Germany). Anti-human TCR αβ-APC antibody (Clone IP26) was purchased from eBioscience™ (Invitrogen, Thermo Scientific, London, UK). Anti-CD3-BV785 (Clone OKT3), anti-CD47-APC (Clone CC2C6), anti-CD69-PerCP (Clone FN50), anti- CD85j (ILT2/LILRB1)-APC (Clone GHI/75), anti-HLA-G-APC (Clone 87G), anti- CD85d (ILT4/LILRB2)-APC (Clone 42D1), anti-mouse TCR β chain-APC (Clone H57-597), anti-CD279 (PD-1)-APC (Clone EH12.2H7), anti-human CD274 (B7-H1, PD-L1)-APC (Clone MIH3) antibodies were purchased from Biolegend (San Diego, CA, USA). Anti-CD8-BV421(Clone RPA-T8) and anti-HLA-A2-APC (Clone BB7.2) were obtained from BD Biosciences. HLA-G blocking antibody based on Tizona Therapeutics Inc (San Francisco, CA, US) patent (WO2020069133A1) was donated by KHAR Medical (Jerusalem, Israel). Lipopolysaccharide (LPS) was purchased from Sigma-Aldrich (Merck, MO, USA) and TGF-β was from Peprotech (Thermo Scientific, London, UK). Premium grade IL-7 and IL-15 were purchased from Miltenyi (Bergisch Gladbach, North Rhine-Westphalia, Germany). Atezolizumab (Tecentriq) was obtained from the Hospital pharmacy.

### 2.2 Cell lines and culture conditions

To validate the E7:E7-TCR interaction in a “universal” manner, a panel of different HLA-A2^+^ (HEK 293T, THP-1, OVCAR-3, A-375, OE-19, MDA-MB-231, and HLA-A2^-^ cancer cells (HT-29, Ramos) cells, together with CaSki (HPV16+, HLA-A2+) and Jurkat were obtained from the American Type Culture Collection (Manassas, VA, USA). 721.221 cells and 721.221-HLA-A2 cells were donated by KAHR Medical (Israel). The CD3 scFv-presenting cell line MDA-MB231-scFvCD3 was generated as previously described (31). Cells were cultured according to the supplier’s recommendation either in RPMI or DMEM (Lonza, Basel, Switzerland) supplemented with 10% fetal calf serum (FCS) (Gibco™ Thermo Fischer Scientific) at 37°C in a humidified 5% CO_2_ atmosphere. Jurkat.NFAT-*Firefly luciferase* (Jurkat*^Fluc^*) cells were purchased from BPS Biosciences (San Diego, CA, US) and cultured in RPMI 10% with 1 mg/mL geneticin (Gibco). Jurkat NFAT-*Gaussia luciferase* (Jurkat*^Gluc^*) cells were obtained after transduction of Jurkat wild type, as explained below (2.6. Lentiviral transductions). All cell lines were regularly tested as free of mycoplasma infection by PCR.

### 2.3 Primary samples

Healthy peripheral blood samples were received as buffy coats from Sanquin, The Netherlands, under agreement number NVT0465.01. Samples were stained for HLA-A2 expression with anti-HLA-A2- APC (Clone BB7.2) before further processing steps were taken as described below.

### 2.4 Isolation of primary T cells and monocyte-derived macrophages

Both primary T cells and monocyte-derived macrophages were obtained from whole blood. First, peripheral blood mononuclear cells (PBMCs) were isolated by density gradient centrifugation with Lymphoprep™ according to the manufacturer’s recommendations (STEMCELL Technologies, Vancouver, BC, Canada). T cells were isolated using an autoMACS Pro Separator (Miltenyi) and the Pan T Cell Isolation Kit (Miltenyi) following the manufacturer’s recommendations. After isolation, T cells were suspended in RPMI with 20% FCS before activation and expansion. To obtain monocyte-derived macrophages, PBMCs were seeded in a 6-well plate at a density of 2.5×10^6^ cells/mL in RPMI 10% FCS containing the M0 differentiation cytokines GM-CSF or M-CSF (50 ng/mL each) for 7 days. To generate M1-type macrophages, M0 cells were treated with LPS (100 ng/mL) and IFN-γ (20 ng/mL) for 1 day. For M2c-type macrophages, M0 cells were primed with IL-10 (50 ng/mL) and TGF-β (50 ng/mL) for 2 days. For experiments, macrophages were harvested from plates using TrypLE Express (Life Technologies, Carlsbad, CA, USA).

### 2.5 T cell activation and expansion

T cells were suspended in TexMacs medium (Miltenyi) containing 12.5 ng/mL of IL-7 and IL-15. T cells were activated overnight with TransAct reagent (Miltenyi) following the manufacturer’s recommendations. After activation, 1x10^6^ cells/condition were used for lentiviral transductions. T cells were cultured in TexMacs media supplemented with IL-7 and IL-15 and expanded for approximately 2 weeks maintaining cell density at 0.5-1x10^6^ cells/mL.

### 2.6 Lentiviral transduction and cloning

Lentivirus was produced by transient transfection of HEK 293T cells, with transfer vector pRRL, psPAX2 and VSV-G packing system using FuGENE (Promega, Madison, WI, USA). Viral supernatants were collected and concentrated using Amicon 100 kDa columns (Merck) according to the manufacturer’s recommendations. Transduction of desired cells, including primary T cells and cell lines, was performed in 12 well-plates by adding 100 µL of concentrated viral supernatant to 0.5-1×10^6^ pre-seeded cells in 1 mL of media. After 48-72h, transduced cells were evaluated for GFP/mCherry expression by flow cytometry and sorted (if needed) with a Sony cell sorter SH800s (Sony Biotechnology, Tokyo, Japan). HPV16-E7 overexpressing cell lines were transduced with pRRL-SFFV-HPV-16-iGFP (Genscript, Nanjing, China) and Jurkat*^Gluc^* were obtained after transduction of Jurkat wt cells with the pRRL-SFFV-NFAT-GLUC_KDEL-PGK-mCherry construct (synthesized by Genscript). KDEL is an ER-retention signal that was introduced to convert the enzyme into a cytoplasmic form and increase the signal read-out magnitude, as previously reported (32). The E7-TCR sequence was described previously as high-avidity HLA-A*02:01-restricted TCR (2) and cloned into the pRRL-SFFV-iGFP plasmid (Genscript) using Gibson cloning assembly. Information about the different PCR experiments can be found in **Table 1**. After DNA purification and ligation, SmaI restriction analysis was used to identify the right construct, followed by sequencing of the pRRL-SFFV-E7-TCR-iGFP (E7-TCR) construct.

**Table 1:** Gibson Cloning PCRs.

PCR	Template	Primers	Product Length	Program
A	pRRL-SFFV-E7_TCR-iGFP	F: GCC ACC ATG GCC CCG GGG CT (20) R: CGG CCA GTA ACG TTA GGG GGG GGG GGC GGA ATT GGG ATC CTT TAT CAT GAA GAC CAG AGC (60)	1900 bp	72°C, 45s
B	pRRL-SFFV-ADGRG1-iGFP	F: GGA TCC CAA TTC CGC CCC CC (20) R: AGT TAA TAG TTT GCG CAA CGT TGT TGC CAT TGC TAC AGG CAT CGT GGT GTC ACG CTC GTC (60)	3900 bp	72°C, 2 min
C	pRRL-SFFV-ADGRG1-iGFP	F: GCC TGT AGC AAT GGC AAC AA (20) R: AAA GCA AGG CCC AAC ACA AAA GCC CCG GGG CCA TGG TGG CGA ATT CCT CGA GAG ATC CGT (60)	3900 bp	72°C, 2 min

### 2.7 E7_11-20_-peptide pulsing assay

HLA-A2 positive cell lines and primary macrophages were seeded overnight in 100 µL of corresponding medium at a density of 10x10^3^ cells/well in 96-well plates. Cells were incubated (pulsed) for 2 h with a final concentration of 10 µg/mL of E7_11-20_-peptide at 37°C and 5% CO_2_ atmosphere before subsequent incubation with 100 µL of *Jurkat ^Fluc/Gluc^*/T cells expressing (TCR+ cells) or not (EV,TCR- cells) the E7-TCR transgene for other 6 h in the case of Jurkat cells or 24-48 h for primary T cells. The effector-to-target (E:T) ratio between Jurkat/T cells and pulsed cells was optimized for each experiment and ranged from 10:1 to 2:1. When required, target cells were pre-treated with 5 µg/mL of blocking antibodies (e.g., anti-HLA-G, Atezolizumab, anti-CD47) for 15 min prior to the addition of the T/Jurkat cells. T cell clustering upon E7_11-20_ pulsing of primary T cells was qualitatively assessed using brightfield microscopy (EVOS Cell Imaging System, Thermo Fischer).

### 2.8 Luminescence assay and quantification

After 6 h incubation with E7-containing cells (E7_11-20_ pulsed or endogenously expressing HPV16), 100 µL of Jurkat NFAT-*Firefly luciferase* (*Jurkat^Fluc^*) or Jurkat NFAT-*Gaussia luciferase* (*Jurkat^Gluc^*) cells were transferred to a white 96-well plate. For *Firefly*, Bio-Glo™ luciferase assay system (Promega, kit #G7941) was added according to the manufacture´s recommendation and incubated for 15 min at RT in the dark. For *Gaussia*, Gluc GLOW assay (Nano Light Technology, Pinetop, AZ, US, #320-50) was added according to the manufacture´s recommendation and incubated for 5 min in the dark. Immediately after, a luminescence readout was performed using a luminescence reader (Synergy, BioTek, Winooski, VT, USA). Relative light unit (RLU) was recorded and analyzed by Gen5 data analysis software (BioTek). Auto-gain was performed, and each condition was measured in triplicate.

For E7_11-20_ pulsing assays, the percentage of increase in RLU (% RLU increase) was calculated in comparison to non-pulsed cells as indicated in formula 1 using the average RLU calculated from triplicate wells. This formula was applied to *Jurkat^Fluc/Gluc^* expressing (TCR+) or not (TCR-) the E7-TCR transgene separately. In this way, a high % RLU increase is expected for TCR+ cells but not TCR-, where the RLU signal with and without peptide is expected to remain constant.


$$\%RLU\;increase=\frac{\left(Average\;RLU\;values_{Pulsed\;cells})-(Average\;RLU\;values_{Non-pulsed\;cells}\right)}{Average\;RLU\;values_{Non-pulsed\;cells}}\times 100$$

*Formula 1. %RLU increase for co-cultures assays with E7-pulsed target cells.*

For calculation on the luminescence increase in target cells expressing HPV16 Formula 2 was used. The % RLU increase was calculated for the TCR+ cells in comparison to the TCR- cells as indicated in Formula 2. In this case, as two different cells are being compared, background correction is initially applied by subtracting RLU produced by Jurkat TCR+ and TCR- alone themselves (at the same cell concentration as each E:T ratio tested).


$$\%RLU\;increase=\frac{\left(Average\;RLU\;values_{TCR+})-(Average\;RLU\;values_{TCR+alone}\right)}{\left(Average\;RLU\;values_{TCR-})-(Average\;RLU\;values_{TCR-alone}\right)}\times 100$$

*Formula 2. %RLU increase for co-cultures with HPV16 expressing target cells with background correction. TCR-/+ alone means only Jurkat cells without target cells.*

After verifying that + TCR and – TCR cells yielded the same background activation levels (**Supplementary Figure A**), for future experiments they were excluded, yielding the final formula as:


$$\%RLU\;increase=\frac{Average\;RLU\;values_{TCR+}}{Average\;RLU\;values_{TCR-}}\times 100$$

*Formula 3. %RLU increase for co-cultures with HPV16-E7 expressing target cells.*

### 2.9 Flow cytometry

Cells were stained for several surface markers expression (e.g., PD-1, PD-L1, HLA-G) by incubating 5x10^3^ cells in 200 µL with corresponding antibodies for 30 min at 4 °C. Cells were washed three times with PBS prior to acquisition in the flow cytometer (CytoFLEX, Beckman Coulter, Brea, CA, USA). Transduction efficiency of Jurkat and primary T cells with the E7-TCR construct was evaluated with a hamster anti-mouse β-TCR chain-APC (clone H57-597, the E7-TCR construct contains murine β chains). Endogenous TCR was measured with anti-human β-TCR-APC antibody (clone H57-597). Upregulation of T cell activation markers was assessed with anti-CD3 BV785, anti-CD8 BV421, anti-CD25-APC and anti-CD69 PerCP5.5. E7-TCR T cells were gated as GFP + cells.

### 2.10 Cytokine production

TNF-α and IFN-γ secretion by primary T cells expressing and lacking the E7-TCR was evaluated by enzyme-linked immunosorbent assay (ELISA). After 24 h co-culture at indicated E:T ratios, the supernatant was harvested, and cytokine concentration was determined using IFN-γ and TNF-α ELISA kits (Immunotools) according to the manufacturer’s instructions.

### 2.11 Quantification of HPV16-E7 mRNA expression

HPV16 expression in CaSki and cell lines lentivirally transduced with HVP16-E7 was evaluated by qRT-PCR. Total RNA was isolated from cells using Rneasy mini kit (Qiagen, Sussex, UK) according to the manufacturer´s instructions. cDNA was synthesized with the iScript™ cDNA Synthesis Kit (Bio-Rad Laboratories, Hercules, CA, US). qRT-PCR was performed using the Sso advanced universal SYBR® green supermix (Bio-Rad) using a standard 40 cycle-program in a thermocycler (CFX384, Bio-Rad). The primers (Genscript) used in the reaction were: AAATGACAGCTCAGAGGAGGAG (forward) and TTTGTACGCACAACCGAAGC (reverse).

### 2.12 Statistical analysis

Significant differences between different samples were evaluated by Student’s t-test. When data from different donors was evaluated in primary T cell experiments, paired Student’s t-test was used. The correlation between HLA-A2 expression and luminescence production was evaluated through a Pearson’s correlation test. Comparison of three or more variables was analyzed by a one-way ANOVA test followed by the *post-hoc* Tukey Kramer test. All tests were performed using GraphPad Prism (GraphPad Prism; GraphPad Software, La Jolla, CA, USA). Where indicated, * = p< 0.05; ** = p< 0.01; *** = p< 0.001.

## 3 Results

### 3.1 HPV16 expressing and E7_11-20_ pulsed cells specifically activate E7-TCR primary T cells

**Figure 1:**
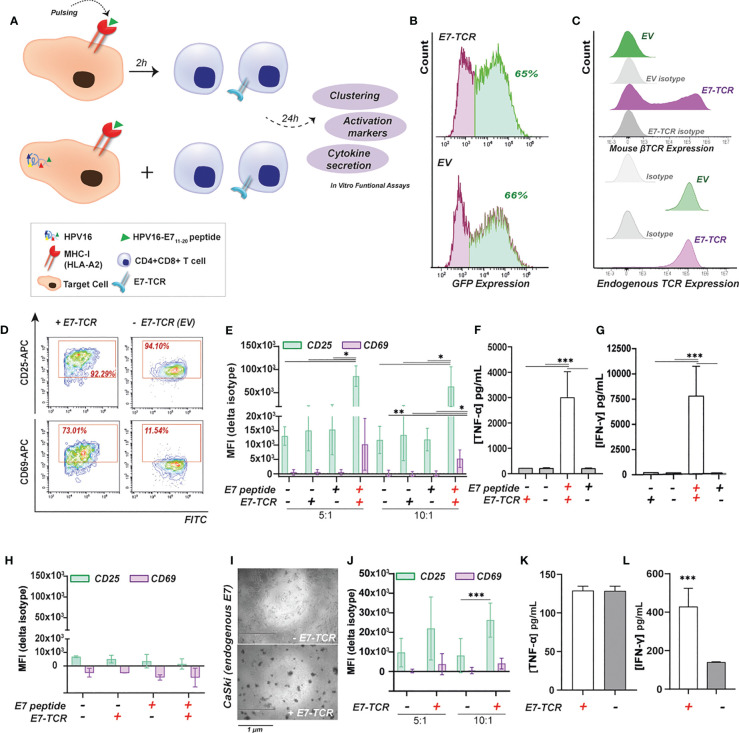
E7_11-20_-specific primary T cell responses using an E7 specific TCR (E7-TCR). **(A)** Target cells expressing or lacking (control) HLA-A2 expression were pulsed with 10 µg/mL of E7_11-20_ peptide. Alternatively, target cells expressing endogenous HPV16, such as CaSki, were also used. Target cells were incubated for 24h with primary T cells expressing E7-TCR or EV at different E:T ratios. Finally, cells as well as supernatants were subjected to different functional assays. **(B)** E7-TCR (above) and EV (below) transduction efficiency measured as % GFP+ cells in a representative T cell donor. **(C)** E7-TCR (above) and endogenous TCR (below) expression within EV and E7-TCR transduced T cells in a representative T cell donor. E7-TCR was measured as mouse β-TCR expression. **(D)** Flow cytometry diagrams for CD25 (APC) and CD69 (APC) upregulation within CD3^+^E7-TCR/EV (FITC)^+^ cells upon 24h co-culture with HEK 293T cells pulsed with 10 µg/mL of E7_11-20_ peptide. **(E)** CD25 and CD69 expression (MFI values) of E7-TCR (+) or EV (-) transduced T cells previously incubated for 24h with HEK 293T cells pulsed with 0 (-) or 10 µg/mL (+) of E7_11-20_ peptide. Results for 5:1 and 10:1 E:T ratios are shown. Mean ± SD, n=3. One-way ANOVA with Tukey’s test for multiple comparison correction **(F)** TNF-α or **(G)** IFN-γ secretion by E7-TCR (+) or EV **(-)** transduced T cells previously incubated with HEK293T cells pulsed with 0 **(-)** or 10 µg/mL (+) of E7_11-20_. Mean ± SD, n=3. One-way ANOVA with Tukey’s test for multiple comparison correction **(H)** CD25 and CD69 expression (MFI values) of E7-TCR (+) or EV (-) transduced T cells previously incubated with HT-29 (HLA-A2-) cells pulsed with 0 (-) or 10 µg/mL (+) of E7_11-20_ Mean ± SD, n=3 **(I)** Microscopy images (EVOS) after 24h co-culture between CaSki cells E7-TCR (+) or EV (- E7-TCR) transduced T cells. **(J)** CD25 and CD69 expression (MFI values) of E7-TCR (+) or EV (-) transduced T cells previously incubated for 24h with CaSki cells. Results for 5:1 and 10:1 E:T ratios are shown. Mean ± SD, n=3, two-sided Student t test **(K)** TNF-α or **(L)** IFN-γ secretion by E7-TCR (+) or EV (- E7-TCR) transduced T cells previously incubated with CaSki cells. Student t test. Where indicated, * = p < 0.05; ** = p < 0.01; *** = p < 0.001.

To set-up and validate the luminescence-based method, a previously described HLA-A2 restricted and HPV16 E7-specific TCR was used (2) using primary T cells and E7_11-20_ pulsed and/or HPV16 E7 expressing target cells as exemplified in **Figure 1A**. Of note, previous studies had used this E7-TCR in combination with E7 _11-19_ peptide instead. Inter-donor variability was observed between T cell batches after lentiviral transduction with the TCR (**Supplementary Figure 1B**), with transduction efficiencies ranging from 45-73% (based on percentage of GFP^+^ cells) for the E7-TCR and from 55-92% for empty vector (EV) control (**Figure 1B**). Staining for the mouse TCR ß-chain confirmed surface expression of the E7-TCR (**Figure 1C**, above), with endogenous TCR expression remaining unaltered (**Figure 1C**, down). After 24 h co-culture of E7-TCR T cells with HEK 293T cells (HLA-A2^+^ model cell line), visual clustering of T cells was detected when target cells had been pulsed with E7_11-20_, but not when peptide and/or E7-TCR were lacking (**Supplementary Material Figure C**). Of note, T cells from HLA-A2^+^ donors already started to cluster in the presence of 10 µg/mL of E7-peptide without E7-TCR, in contrast to T cells from HLA-A2^-^ donors (**Supplementary Material Figure D**). Therefore, all the TCR-E7 experiments reported here were performed with HLA-A2^-^ donors. E7_11-20_-specific T cell activation was confirmed by the selective up-regulation of CD25 (6 ± 0.6 and 3 ± 0.7-fold change for E:T of 5.1 and 10.1, respectively) and CD69 (20 ± 1 and 7 ± 1.5-fold change for E:T of 5.1 and 10.1, respectively) in E7-TCR T cells co-cultured with E7_11-20_-pulsed HEK 293T cells (**Figures 1D, E**). Gating strategy and flow diagrams for the upregulation of CD25 and CD69 for EV T cells are shown in **Supplementary Material Figures E, F**. Additionally, when mixed with E7_11-20_-pulsed HEK 293T cells, E7-TCR T cell secreted significantly higher levels of TNF-α (**Figure 1F**) and IFN-γ (**Figure 1G**). In contrast, no such activation was detected upon mixed culture of E7-TCR cells with the HLA-A2^-^ cell line HT-29 (**Figure 1H**, **Supplementary Material Figure G**). Thus, the E7-TCR T cells selectively reacted to cells presenting E7_11-20_ peptide: MHC-I complexes (E7pMHC).

Next, the ability of E7-TCR T cells to be activated by endogenously processed HPV16 was investigated using CaSki cells (as shown in **Figure 1A**). CaSki cells endogenously express HPV16, as validated by RT-qPCR (**Supplementary Material Figure H**). After 24h of mixed culture of CaSki and E7-TCR T cells, a clear T cell clustering was observed, whereas control EV T cells did not (**Figure 1I**). In these mixed cultures with CaSki, CD25 was significantly upregulated in E7-TCR cells compared to EV T cells by 2 ± 0.14 and 5.5 ± 3.50-fold change for E:T ratios of 5.1 and 10.1, respectively (**Figure 1J**). However, not significant difference was observed for CD69 upregulation at any E:T ratio. In terms of cytokine secretion, only a significant increase in IFN-γ and not TNF-α secretion was detected (**Figures 1K, L**). Altogether, this data confirmed the specificity of the E7-TCR molecule to induce E7_11-20_-specific T cell responses *in vitro* after both E7_11-20_ pulsing of target cells and upon endogenous HPV16 processing and E7_11-20_ presentation in MHC-I.

### 3.2 E7_11-20_ peptide specifically activates Jurkat.NFAT-*Fluc* E7-TCR cells, leading to antigen-specific luminescence production

**Figure 2:**
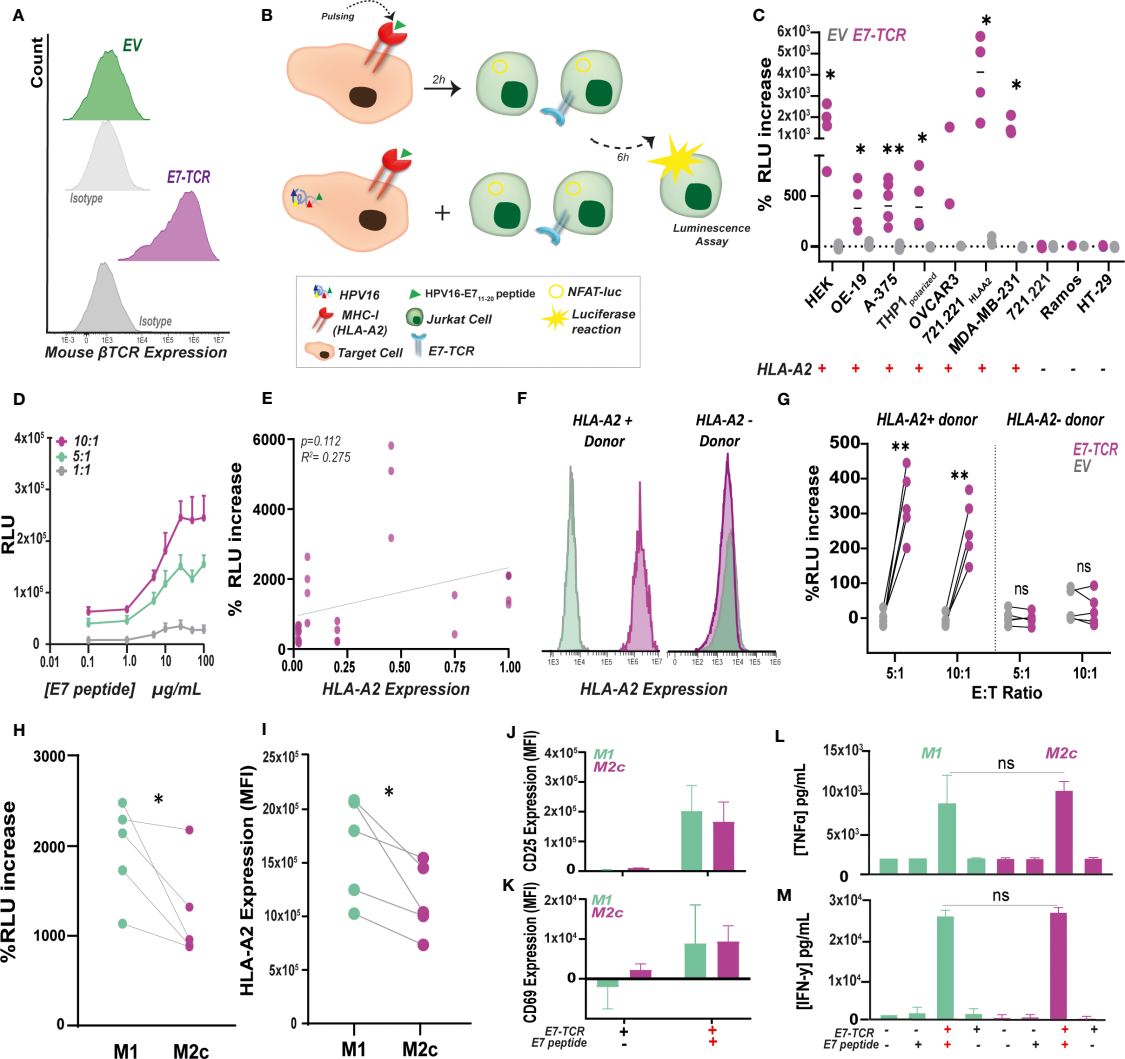
E7-specific luminescence production as a rapid alternative to measure antigen-specific T cell responses *in vitro*. **(A)** E7-TCR expression in Jurkat.NFAT-*FLuc* (Jurkat^Fluc^) cells measured as mouse β-TCR expression by flow cytometry. **(B)** Target cells pulsed with the E7_11-20_peptide or endogenously expressing HPV16 were incubated at different E:T ratios with Jurkat.NFAT-*Fluc* cells expressing (Jurkat*^Fluc.E7-TCR^*) or lacking (Jurkat*^Fluc.EV^*) the E7-TCR construct. After 6h incubation, BioGlow solution was added, and luminescence was measured as relative light units (RLU). **(C)** Percentage of RLU increase between E7_11-20_-pulsed (10 µg/mL) and non-pulsed cells was evaluated in a panel of several HLA-A2^+^ (HEK 293T, OE-19, A-375, THP-1, OV-CAR-3, 721.221HLA-A2, MDA-MB-231) and HLA-A2^–^ (Ramos, HT-29, 721.221) cell lines. %RLU increase was calculated for Jurkat*^Fluc.E7-TCR^* (purple) and Jurkat*^Fluc.EV^* (gray) as indicated in Formula 1. n>3, Student’s t-test, with p<0.05 significant. **(D)** Dose-response curve upon pulsing of MDA-MB-231 cells with increasing E7_11-20_ concentrations. Resulting RLU units are shown for 10.1 (purple), 5.1 (green) and 1.1 (gray) Jurkat^Fluc.E7-TCR^ cells. Mean ± SD, n=3 **(E)** Correlation between HLA-A2 expression in target cells and %RLU increase by Jurkat*^Fluc.E7-TCR^* cells obtained on figure **(C)** using Formula 1. Linear regression p=0.112. Pearson`s r=0.275. **(F)** HLA-A2 expression in CD11b^+^ PBMCs within two representative HLA-A2 positive and negative donors. **(G)** Percentage of RLU increase between E7_11-20_-pulsed (10 µg/mL) and non-pulsed monocyte-derived macrophages (both M1 and M2c) obtained from HLA-A2 positive (n=5) and negative (n=5) donors. %RLU was calculated for Jurkat*^Fluc.E7-TCR^* (purple) and Jurkat*^Fluc.EV^* (gray) as indicated in Formula 1. E:T ratios of 5:1 and 10:1 were used. Paired student’s t-test, with ** = p < 0.01. **(H)** Percentage of RLU increase calculated for Jurkat*^Fluc.E7-TCR^* between E7_11-20_-pulsed (10 µg/mL) and non-pulsed M1 (n=5) and M2c (n=5) macrophages using Formula 1. Student’s t-test, with p= 0.046. **(I)** HLA-A2 expression indicated as MFI values in M1 and M2c-monocyte derived macrophages from the same donor. Student’s t-test, with p=0.032. CD25 **(J)** and CD69 **(K)** expression indicated as MFI values in primary T cells expressing the E7-TCR previously co-culture for 6h with M1 and M2c macrophages pulsed (+) or non-pulsed (-) with the E7_11-20_. n=3 T cells batches. TNF-α **(L)** and IFN-γ **(M)** secretion by primary T cells expressing (+) or lacking (-) the E7-TCR co-culture for 6h with M1 and M2c macrophages pulsed (+) or non-pulsed (-) with E7_11-20_ n=3 T cells batches. Where indicated, * = p< 0.05; ** = p< 0.01.

To establish a versatile reporter for evaluating TCR activation, Jurkat.NFAT-*Firefly luciferase* cells (defined as Jurkat^Fluc^) were transduced with the E7-TCR (Jurkat^Fluc.^*^E7-TCR^*) or EV (Jurkat^Fluc.^*^EV^*) construct and sorted based on GFP signal. Within this cell line, >90% of E7-TCR cells expressed the mouse *β-*TCR chain (**Figure 2A**). Notably, the threshold for Jurkat activation as well as the magnitude of the luminescence response might differ from primary T cells (33). Therefore, the specificity and window for measuring E7-TCR signaling, using luminescence as read-out, were first delineated (for schematic see **Figure 2B**). Hereto, a panel of cell lines with high (CaSki, HEK 293T, THP-1, OVCAR-3, MDA-MB-231, 721.221^HLA-A2^), low (OE-19, A-375) and no expression of HLA-A2 (721.221, Ramos, HT-29) (**Supplementary Material Figure I**) was used. Upon co-culture with Jurkat^Fluc.^*^E7-TCR^*, luminescence was significantly increased by all the HLA-A2^+^ E7_11-20_ pulsed cell lines compared to non-pulsed cell lines (**Figure 2C**, for raw RLU values **Supplementary Material Figure J**) Signaling induced by E7-TCR was dependent on dose of peptide and E:T ratio, with an EC50 at 6.5 µg/ml peptide in E:T ratio 10:1 (**Figure 2D**, **Supplementary Material Figure K**). In contrast, no increase in luminescence was detected in co-cultures with HLA-A2^-^ cell lines (**Figure 2C**), nor upon incubation of Jurkat^Fluc.EV^ cells with HLA-A2^+^ cells (**Figure 2C**). Of note, within this cell line panel the level of HLA-A2 did not significantly correlate with the increase in luminescence (**Figure 2E**, Pearson´s r= 0.275).

Next, monocyte-derived macrophages from HLA-A2^+^ and HLA-A2^-^ donors were used to evaluate this system in the context of professional antigen presenting cells (**Figure 2F**). In line with expectations, E7_11-20_ pulsed-HLA-A2^+^ macrophages triggered a significant increase in luminescence in Jurkat^Fluc.^*^E7-TCR^* compared to non-pulsed cells (**Figure 2G**). Further, using this system, E7_11-20_ pulsed and M1-like differentiated macrophages induced significantly higher TCR activation and luminescence than M2-like differentiated macrophages obtained from the same donor (**Figure 2H**). This finding is in line with the significantly higher HLA-A2 expression detected in M1-like differentiated macrophages (**Figure 2I**). Notably, when the same macrophages were co-cultured with primary E7-TCR-transduced T cells, no significant difference between macrophage subtype in T cell activation was detected as defined by CD25 (**Figure 2J**) and CD69 (**Figure 2K**) expression. Furthermore, secretion of TNF-α (**Figure 2L**) and IFN-γ (**Figure 2M**) by E7-TCR T cells did not significantly differ between M1 and M2c macrophages. Thus, E7-specific induction of luminescence by Jurkat^Fluc.^*^E7-TCR^* cells can be used to monitor E7-TCR activation by E7_11-20_ peptide:MHC-I complexes and can be used to investigate M1 and M2 effects on antigen presentation.

### 3.3 Immunosuppressive checkpoints reduced antigen-specific luminescence production by Jurkat^Fluc.E7-TCR^ cells, an effect that could be abrogated by corresponding antagonistic antibodies

**Figure 3:**
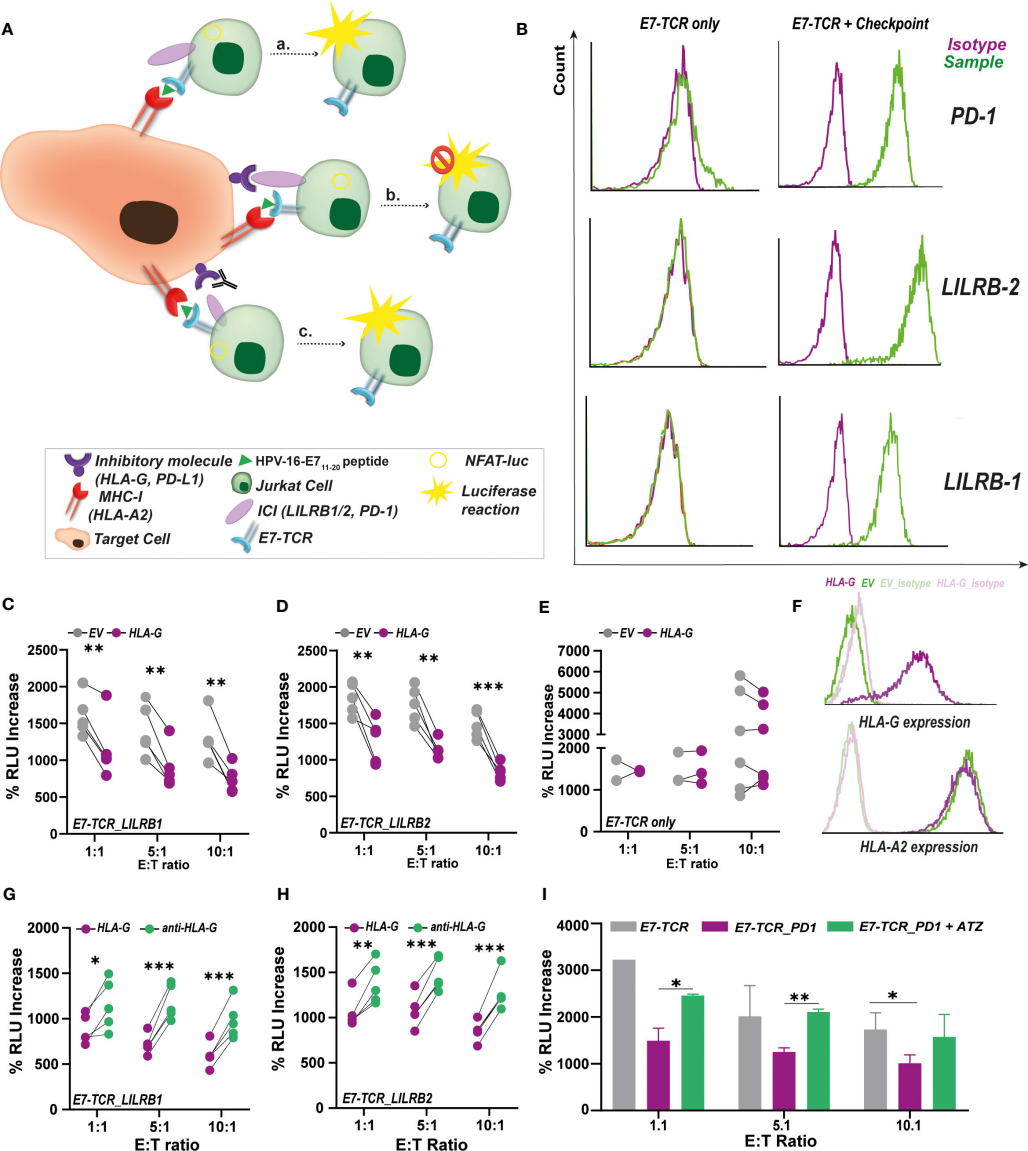
Impact of immunosuppressive checkpoints on E7_11-20_/E7-TCR mediated antigen-specific luminescence production. **(A)** Pulsing of target cells with E7_11-20_ peptide was used to study the inhibitory impact of several immunosuppressive checkpoints (including HLA-G and PD-L1) on T cell activation. For that, target cells expressing HLA-G were co-culture with Jurkat^Fluc.E7-TCR^ cells expressing LILRB-1 (Jurkat ^LILRB-1^) and LIRLB-2 (Jurkat^LILRB-2^), interacting partners of HLA-G in T cell mediated inhibition. Alternatively, Target cells expressing PD-L1 were also co-cultured with Jurkat.NFAT-luc_E7-TCR cells expressing PD-1 (Jurkat^PD-1^). As consequence of these inhibitory signals, T cell activation and concomitant luminescence production is expected to be decreased. However, disruption of these inhibitory signals by targeting therapeutic antibodies (e.g., anti-HLA-G, anti-PD-L1 (Atezolizumab)) T cell activation and luminescence production would be restored. **(B)** Surface expression of PD-1, LILRB-1 and LILRB-2 in modified Jurkat^Fluc.E7-TCR^ cells (right) compared to E7-TCR only (left). Percentage of RLU increase between E7_11-20_-pulsed (10 µg/mL) and non-pulsed 721.221^HLA-A2^ cells expressing HLA-G (purple) or EV (gray) calculated for Jurkat ^LILRB-1^ **(C)** and Jurkat ^LILRB-2^ **(D)** cells. Jurkat were added at E:T ratios of 1:1 (n=5), 5:1 (n=5) and 10:1 (n=5). Student’s t-test, with p<0.05 significant. **(E)** Percentage of RLU increase between E7_11-20_-pulsed (10 µg/mL) and non-pulsed 721.221.^HLA-A2^ cells expressing HLA-G (purple) or EV (gray) calculated for Jurkat^Fluc.E7-TCR^ only. Jurkat were added at E:T ratios of 1:1 (n=2), 5:1 (n=3) and 10:1 (n=6). Student’s t-test, with p-value non-significant. **(F)** Surface HLA-G (up) and HLA-A2 (down) expression in 721.221^HLA-A2^ cells expressing (purple) or lacking (green) HLA-G. HLA-G is only expressed in HLA-G expressing cells while equal HLA-A2 expression was found in both HLA-G and EV cells. Percentage of RLU increase between E7_11-20_-pulsed (10 µg/mL) and non-pulsed 721.221^HLA-A2^ HLA-G+ cells untreated (purple) or treated with 5 µg/mL of anti-HLA-G (green) calculated for Jurkat ^LILRB-1^ **(G)** and Jurkat ^LILRB-2^ **(H)** cells. Jurkat were added at E:T ratios of 1:1 (n=5), 5:1 (n=5) and 10:1 (n=5). Student’s t-test, with p<0.05 significant. **(I)** Percentage of RLU increase between E7-pulsed (10 µg/mL) and non-pulsed MDA-MB-231 cells calculated for Jurkat^Fluc.E7-TCR^ only (gray) or for Jurkat ^PD-1^cells. Luminescence production is decreased due to the inhibitory effect of PD-1/PD-L1 interaction. However, when MDA-MB-231 cells were pre-treated with 5 µg/mL of anti-PD-L1 antibody Atezolizumab, luminescence production by Jurkat ^PD-1^(green) could be restored. n=3, Student’s t-test, with p<0.05 significant. %RLU increase was calculated in all cases according to Formula 1. Where indicated, * = p< 0.05; ** = p< 0.01; *** = p< 0.001.

To validate the utility of the TCR-E7 reporter system, E7_11-20_-specific T cell responses were evaluated in the context of several established immunosuppressive interactions with implications in T cell activation (**Figure 3A**), including HLA-G/LILRB-1,2 (34) and PD-1/PD-L1 (35) signaling. Jurkat^Fluc.^*^E7-TCR^* cells were modified to express the inhibitory checkpoints LILRB-1 (Jurkat*^LILRB-1)^*, LILRB-2 (Jurkat*^LILRB-2^)* or PD-1 (Jurkat*^PD-1^)*, with parental Jurkat^Fluc.^*^E7-TCR^* lacking expression of these checkpoints (**Figure 3B**). Co-culture of Jurkat*^LILRB-1^* cells with E7_11-20_ pulsed 721.221^HLA-A2^ yielded a significant lower % of increase in RLU when these target cells expressed the inhibitory molecule HLA-G (**Figure 3C**). Similar results were obtained with Jurkat*^LILRB-2^* cells (**Figure 3D**). Accordingly, HLA-G expression on 721.221^HLA-A2^ did not impact the % RLU increase by parental Jurkat^Fluc.^*^E7-TCR^* cells that lacked this checkpoint (**Figure 3E**). Importantly, both HLA-G and EV 721.221^HLA-A2^ cells expressed equal levels of HLA-A2 (**Figure 3F**), confirming that the negative impact observed in T cell activation was due to immunosuppressive signaling induced by HLA-G/LILRB-1,-2 interaction. In line with that, when HLA-G^+^ cells were pre-treated with 5 µg/mL of an HLA-G blocking antibody, T cell activation by Jurkat*^LILRB-1^* (**Figure 3G**) and Jurkat*^LILRB-2^* cells (**Figure 3H**) was restored, yielding a significant % of increase in RLU. As control, binding of anti-CD47 antibody InhibRx (5 µg/mL), containing the same IgG isotype (IgG4) as the anti-HLA-G antibody, did not have any impact on T cell activation, supporting the specificity of the anti-HLA-G antibody treatment (**Supplementary Material Figure L**). For the PD-1/PD-L1 checkpoint, incubation of Jurkat*^PD-1^* cells with E7_11-20_ pulsed MDA-MB-231 cells (HLA-A2^+^, PD-L1^+^) yielded significant lower % increase in RLU than parental Jurkat cells lacking PD-1 (**Figure 3I**). T cell activation was significantly increased upon treatment of MDA-MB-231 cells with 5 µg/mL of Atezolizumab (ATZ, anti-PD-L1 antibody) (**Figure 3I**). Of note, Jurkat^Fluc.^*^EV^* did not trigger any increase in RLU upon co-culture with any of the E7_11-20_ pulsed cell lines in these experiments (**Supplementary Material Figure M**). Altogether this data supports the suitability of this assay for rapid and facile evaluation of the immunosuppressive tumor microenvironment including inhibitory molecules (e.g., novel checkpoints) in triggering antigen-specific T cell responses, as well as the impact of targeted therapeutics.

### 3.4 E7_11-20_/E7-TCR specific reporter system performed similarly as non-TCR specific anti-CD3 luciferase-based method for Jurkat activation assessment

**Figure 4:**
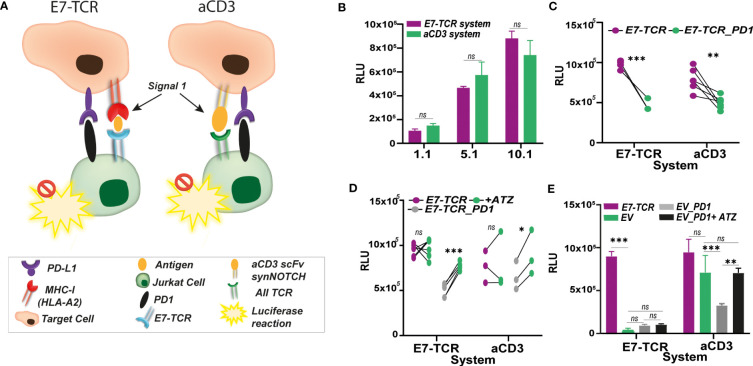
E7-TCR system performance compared to anti-CD3 approaches. **(A)** Contrary to anti-CD3 systems, in which anti-CD3 binding structures (e.g., anti-CD3 scFv) are present in the target cells to induced Signal 1 and trigger a non-specific TCR-mediated T cell activation, the E7-TCR based system specifically activates T cells containing the antigen-specific TCR through its interaction with antigen: MHC complexes. In both systems, the presence of inhibitory molecules such as PD-1/PDL1 might hamper T cell activation which can be easily measured by luminescence production using Jurkat.NFAT-Fluc (Jurkat^FLuc^) cells. However, the antigen-specific system (here E7-TCR) provides a more realistic setting of the natural mechanism for T cells activation. **(B)** Relative light units (RLU) measured upon co-culture between Jurkat^Fluc.E7-TCR^ cells with MDA-MB-231 (HLA-A2^+^) previously pulsed with E7 peptide (described as E7-TCR system, purple) or MDA-MB-231^aCD3scFv^ (described as aCD3 system, green). Similar values of RLU were obtained though both systems, despite E7-TCR system induced activation only through the E7-TCR. n=3 **(C)** Relative light units (RLU) measured upon co-culture between Jurkat^Fluc.E7-TCR^ cells (purple) and Jurkat^.PD-1^cells (green) with MDA-MB-231 (HLA-A2^+^) previously pulsed with E7 peptide (described as E7-TCR system, purple, n=3) or MDA-MB-231^aCD3scFv^ (described as aCD3 system, green, n=5). In both cases, PD-L1/PD-1 interaction induced decrease RLU values. Paired t test, with p< 0.05 significant. **(D)** Relative light units (RLU) measured upon co-culture between Jurkat^Fluc.E7-TCR^ cells (purple), Jurkat^PD-1^cells (gray) with MDA-MB-231 (HLA-A2^+^) previously pulsed with E7 peptide (described as E7-TCR system) or MDA-MB-231^aCD3scFv^ (described as aCD3 system) alone or previously treated with 5 µg/mL of anti-PD-L1 antibody Atezolizumab (ATZ) (green). ATZ treatment restored luminescence production only by Jurkat^PD-1^cells but not E7-TCR alone. **(E)** Relative light units (RLU) measured upon co-culture between Jurkat^Fluc.E7-TCR^ cells (purple), Jurkat^Fluc.EV^ (green), Jurkat^Fluc.EV.PD-1^ (gray) with MDA-MB-231 (HLA-A2^+^) previously pulsed with E7 peptide (described as E7-TCR system) or MDA-MB-231^aCD3scFv^ (described as aCD3). Also, RLU resulting from Jurkat^FLuc.EV.PD-1^ incubation with cells pre-treated with 5 µg/mL ATZ (black) is shown. Jurkat^Fluc.EV^ with or without PD-1 was observed only in aCD3 system. Where indicated, ns, non-significant; * = p< 0.05; ** = p< 0.01; *** = p< 0.001.

Performance of the E7-TCR system was compared to another luciferase-based method previously used by us and others to measure Jurkat activation in a TCR-dependent way. This method is based on the presence of an anti-CD3 (aCD3) scFv on the target cells, leading to activation of TCR in a non-antigen specific manner (**Figure 4A**) and yields high luminescence signals. Co-culture between MDA-MB-231 (PD-L1^+^) cells expressing the aCD3-scFv (MDA-MB-231^aCD3-scFv^) and Jurkat.NFAT-F*luc* E7-TCR (Jurkat^Fluc.^*^E7-TCR^)* cells induced similar levels of RLU as the E7_11-20_ specific activation upon peptide loading (**Figure 4B**). Both systems were also comparable in terms of measuring the impact of the immunosuppressive PD-1/PD-L1 interaction in Jurkat activation (**Figure 4C**), as well as the impact of atezolizumab treatment (**Figure 4D**). However, whereas MDA-MB^231aCD3-scFv^ induced a non-TCR specific activation of the Jurkat cells, E7_11-20_ pulsed MDA-MB-231 specifically activated Jurkat^Fluc.^*^E7-TCR^*, as exemplified by the activation of Jurkat^Fluc.^*^EV^* cells only by the aCD3 scFv system (**Figure 4E**). In line with this, impact of PD-1/PD-L1 interaction could also be measured using Jurkat*^Fluc.EV^* cells expressing PD-1 only with MDA-MB-231^aCD3-scFv^ cells (**Figure 4E**).

### 3.5 Endogenous HPV16 processing and presentation leads to lower but still detectable antigen-specific bioluminescence production by *Firefly and Gaussia luciferase* reporter Jurkat cells

**Figure 5:**
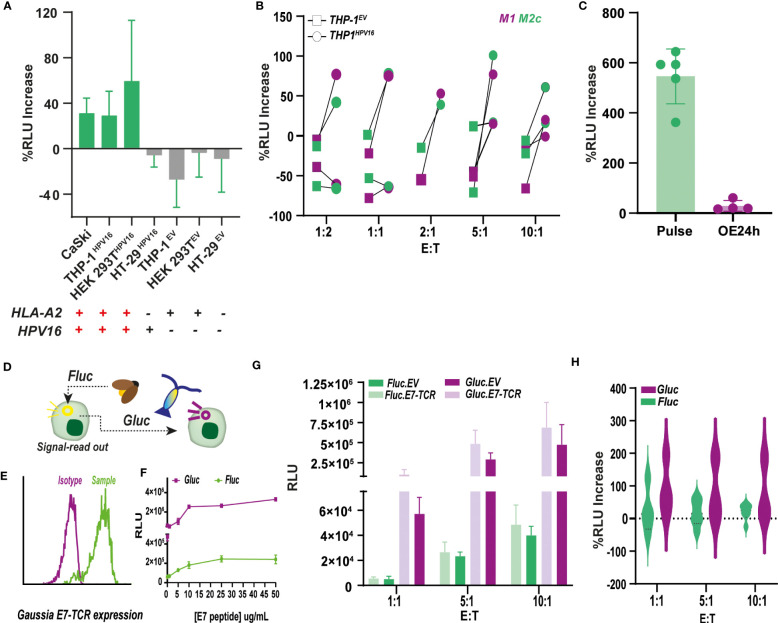
Antigen-specific bioluminescence production by Firefly and Gaussia luciferase reporter Jurkat cells upon endogenous HPV16-E7_11-20_ presentation **(A)** Percentage of increase in relative luminescence units (RLU) of Jurkat^Fluc.E7-TCR^ cells upon co-culture with a panel of cell lines with different HLA-A2 and HPV16 expression. % Increase of RLU was calculated respect to E7-TCR lacking cells according to Formula 3. n<3. **(B)** Percentage of RLU increase by Jurkat^Fluc.E7-TCR^ cells upon co-culture with polarized THP-1 cells expressing (circle) or lacking (squares) HPV16 expression. THP-1 was polarized towards M1 (purple) and M2c (green) macrophages, although no significant difference between subtypes was observed.% Increase of RLU was calculated respect to E7-TCR lacking cells according to Formula 3. n<3. **(C)** Comparison between the % of RLU increase by Jurkat^Fluc.E7-TCR^ cells upon co-culture with polarized THP-1 cells previously pulsed with 10 ug/mL of E7_11-20_ peptide (defined as E7 Pulse, green, n=5) or expressing HPV16 (defined as HPV16 OE, purple, n=4). Student’s test with p<0.0001 **(D)** NFAT.*Firefly luciferase* (*FLuc*) reporter system was replaced by NFAT.*Gaussia luciferase* (*Gluc*), an enzyme with described higher sensitivity. **(E)** E7-TCR expression in Jurkat.NFAT-*Gluc* (Jurkat^Gluc^) cells measured as mouse β-TCR expression by flow cytometry. **(F)** Dose-response curve upon pulsing of MDA-MB-231 cells with increasing E7_11-20_ peptide concentrations. Resulting RLU units are shown for 10.1 E:T for Jurkat^FLuc.E7-TCR^ (green), and Jurkat ^Gluc.E7-TCR^ (purple) **(G)** RLU produced by Jurkat.NFAT Fluc (green) and Gluc (purple) expressing (light) or lacking (dark) the E7-TCR construct. Jurkat were added at E:T ratios of 1:1 (n=3), 5:1 (n=3) and 10:1 (n=3). **(H)** Comparison of % RLU increase obtained for E7-TCR Jurkat cells at different E:T when Gaussia (Gluc, purple) or Firefly (Fluc, green) reporter cells were used. With some variability in the data, no significant differences were observed.

The ability of Jurkat^Fluc.^*^E7-TCR^* to produce antigen-specific luminescence upon, not only E7_11-20_ pulsing, but also endogenous antigen processing and presentation is crucial to proceed with further applications of the method. In this regard, co-culture of CaSki and other HPV16 expressing cell lines, including THP-1, HT-29 and HEK 293T, with Jurkat^Fluc.^*^E7-TCR^* cells, a small increase (20-50%) (yet not significant) in RLU compared to Jurkat^Fluc.^*^EV^* was obtained for HLA-A2^+^ cells (**Figure 5A**). Also, no significant differences were observed between M1 and M2c polarized THP1^HPV16+^ cells (**Figure 5B**), this in contrast to the above-described primary M1 and M2c polarized macrophages. Importantly, as illustrated for THP-1 (10:1 E:T), the magnitude of the luminescence signal was more than 20 times lower than that obtained upon direct E7_11-20_ peptide pulsing (**Figure 5C**).This, together with the lack of statistical significance of previous experiments highlighted the need to expand the measurement window of the assay. When an increase in sensitivity was explored by exchanging standard *Firefly luciferase (Fluc)* by *Gaussia luciferase (Gluc)* (**Figures 5D, E**), which yielded a 3-4 fold increase in RLU values upon Jurkat activation by CD3/CD28 Dynabeads (**Supplementary Material Figure N**) and E7_11-20_ pulsed MDA-MB-231 (HLA-A2^+^) cells (**Supplementary Material Figures O, P**). Jurkat^Gluc^ cells expressing the E7-TCR (Jurkat^Gluc.^*^E7-TCR^)* had an EC50 of 7.9 ug/mL peptide for E:T of 1:10, with a 1 magnitude fold-increase in RLU compared to the Firefly counterpart (**Figure 5F**). Co-culture of CaSki with Jurkat^Gluc.^*^E7-TCR^* and Jurkat^Gluc.^*^EV^* cells led to significantly higher values of RLU compared to their *Firefly* counterparts (**Figure 5G**). However, due to a higher background signal of Jurkat^Gluc.^*^EV^* (**Figure 5G**), no significant difference between Fluc and Gluc systems was obtained when comparing the % of RLU increase between E7-TCR and EV cells (**Figure 5H**). Similar results were obtained for co-culture of polarized THP-1^HPV16+^ with Gluc cells (**Supplementary Material Figure Q**).

## 4 Discussion

Here, we describe a versatile antigen-specific Jurkat reporter system to monitor immunomodulation in the context of antigen-specific T cell responses *in vitro*. Combining the HPV16 E7_11-20_ peptide and Jurkat.NFAT-*luc* cells engineered with a high-avidity E7_11-20_-specific TCR (E7-TCR), the system has allowed for the detection of antigen-specific luminescence production due to the specific recognition of E7_11-20_ peptide:MHC-I complexes. The system also allowed for the detection of specific responses upon endogenous HPV16 processing and presentation, although further optimization of the magnitude of the signal-readout might be desirable for future applications. Overall, this method is suitable for the rapid study of antigen-specific T cell responses within the immunotherapy field and may be implemented to facilitate the design and development of novel therapeutic strategies.

The Jurkat E7-TCR reporter system described here was able to quantify the impact of immune checkpoints and their cognate inhibitors on the magnitude of E7 T cell responses. Similarly, this system was able to detect the impact of M1 and M2 macrophage differentiation. Thus, the system can be used to screen for the impact of immunomodulation on specific immune responses. Notably, although TCR equipped Jurkat reporters have been previously described, in the context of TCR screens, the screening of immunomodulation has so far been performed with unspecific activation of TCR signaling, e.g. mediated by CHO cells ectopically expressing anti-CD3 molecules (CHO/aCD3) (36, 37). Such non-specific TCR triggering of therapeutic antibodies targeting several checkpoints, such as TIGIT (17), PD-L1 (38), LAG-3 (39) has been extensively validated. Here, we show that the response of the Jurkat E7-TCR reporter system was comparable in magnitude to the one obtained through the standard CHO/aCD3 system, while offering a more physiological mechanism of triggering T cell activation in an antigen-specific manner and negating the need to manipulate target cells with the anti-CD3 scFv.

Several approaches, relying on both luminescence and fluorescence, have been described for the assessment of antigen-specific T cell activation *in vitro* using Jurkat reporter systems (12–14). For instance, by using a fluorescence reporter Jurkat 76 T cell line, simultaneous NF-kB, NFAT and AP-1 activation was evaluated upon antigen-specific responses against tumor and virus antigens (7, 8). Similarly, another study reported the screening of functional CMV specific T-cell receptors by using a Jurkat NF-kB GFP reporter system. In the former study, incorporation of adhesion molecules (CD2/CD226) and/or co-stimulatory molecule CD80 was required to increase the percentage of NFAT positive reporting cells upon antigen pulsing in HLA corresponding target cells above 50% (8). Despite being a promising strategy to enhance the system’s sensitivity, these manipulations also increased the background signal. This net background is characteristic of fluorescent assays as a consequence of the high influx of photons during the excitation state (11). Although luminescence typically yields lower light intensities to the slower rate by which the excited states are created, the assay backgrounds are highly reduced and therefore, luminescence-based assays as the one described here, can provide up to 10- to 1,000 fold higher assay sensitivity than fluorescence assays (40, 41).

Compared to peptide-pulsed target cells, the luminescence signal obtained upon co-culture of the Jurkat E7-TCR reporter cells with HPV16 expressing cells (e.g., CaSki) was decreased 11-fold. The expression of HPV16 E7 oncoprotein has been previously associated with reduced IFN-y mediated MHC-I presentation (42–44). Although we haven’t investigated this in our HPV16-E7 overexpressing cell lines, at least in CaSki, neither HLA-A2 nor IFN-γ production was altered. This reduced sensitivity is also in line with the lower expected number of E7_-_pMHC copies upon endogenous presentation. In fact, previous studies using similar antigen-specific Jurkat reporter cells also described lower luminescence production due to insufficient expression of cognate antigen (12). Methods for absolute quantification of pMHC (copies/cell) are very limited and results will depend on several factors such as the peptide, number of MHC complexes or cell type (45), but 25 copies of E7_11–19_:MHC per cell have been reported for CaSki (46). Thus, the Jurkat system described here is sufficiently sensitive to pick up these low pMHC numbers, with a 4-10-fold increase in luminescence. Notably, co-culture of CaSki with primary transgenic E7-TCR T cells also yielded a clear T cell activation (illustrated by at least 2-fold increase in CD25 and CD69 expression and significant higher IFN-γ secretion), highlighting the higher sensitivity of primary T cells compared to Jurkat reporter cells. This sensitivity difference is in line with the reported distinct threshold for cell activation between primary T cells and Jurkat, mainly due to differences in their kinase activity profile and predominant signaling pathways (33).

In order to increase the sensitivity of the Jurkat reporter cells, *Firefly luciferase* was exchanged by *Gaussia luciferase*, which had a reported higher sensitivity of ~40 fold (32). In fact, the magnitude of the response in the current system was increased, but only by up to 4-fold. However, this increase in signal was accompanied with a considerably higher background signal, which did not allow for a real improvement in the analysis. Alternative strategies, including the incorporation of the above-mentioned CD2/CD226 adhesion molecules or CD8 co-receptor molecule to Jurkat (which is a CD4+ cell line), might be considered to further increase the sensitivity of the response upon endogenous HPV16 presentation. In line with this, CD8 expression increased the fluorescence reporter signal upon NFAT and NF-kB activation in a Jurkat E6.1 cell system (10), and was associated with lower sensitivity in Jurkat NFAT-luc cells (12). Another important parameter that might affect the method’s sensitivity is the avidity of the TCR itself since the strength of T cell responses correlates with TCR avidity/affinity (47). With the E7-TCR being reported as a high-avidity TCR (2), lower limit of detections could be defined for different avidity TCRs. However, the lower threshold for activation or sensitivity observed for the E7-TCR Jurkat over primary T cells was found to have some advantages. For instance, only through the E7-TCR Jurkat system, but not with transgenic primary T cells, were significant differences between M1 and M2c macrophages observed in triggering T cell activation. Thus, the E7-TCR Jurkat system might be a helpful tool not only to evaluate antigen-specific responses *in vitro*, but also to provide a better window for the evaluation of the impact of novel (inhibitory/stimulatory) molecules or therapeutics on T cell responses *in vitro*.

Interestingly, the specific response to E7_11-20_ peptide did not correlate with HLA-A2 expression levels when pulsed on a panel of several HLA-A2^+^ cell lines. However, the binding of extracellular peptides to cell surface MHC is not sufficiently well understood to predict the number of pMHCs formed on cells after exposing them to an extracellular peptide. Notably, the E7_11-20_ peptide is described to have a higher binding affinity for HLA-A2*(02:01) molecules (2), but can have some cross reactivity with other HLA-A2*02 alleles as previously reported (46). Such allelic variability may have impacted on E7_11-20_ peptide presentation to E7-TCR within the different HLA-A2^+^ cell lines. Several studies have described that protein antigen expression does not necessarily correlate with peptide:MHC complex density (48, 49). In our experiments the same concentration of antigen was pulsed, thereby avoiding any influence of the intracellular antigen presentation machinery. HLA-A2^+^ cell lines might also have different ratios of vacant peptide-binding sites on the MHC class I molecules on their cell surface (50), which could be directly correlated with amount of E7_11-20_ loading and the signal readout measured. On the other hand, pulsing of M1 macrophages yielded significantly higher Jurkat activation than M2c, a fact that coincided with and could be related to the higher HLA-A2 expression found in M1 macrophages. Interestingly, although some studies have highlighted the superior ability of M1 macrophages to promote antigen presentation over M2c (51), evaluation of HLA-A2 expression between M1 and M2c is lacking in literature. The current results highlight the potential relevance of MHC expression levels in polarized macrophage responses and warrant follow-up studies to delineate whether differential activation is due to HLA-A2 expression or differential antigen processing.

Finally, this method has potential as a predictive tool for MHC-dependent “off-target” effects in drug development, as previously described (14) or for early detection and management of immune-related adverse events (irAEs) in combination with bioluminescence imaging (BLI) (52). As E7-TCR Jurkat would produce luciferase upon antigen-specific activation, real-time, spatial information on T-cell infiltration, persistence, and anti-tumor activity in visceral organ sides may be obtained (53, 54). Also, further development of these systems might be useful for evaluating endogenous antigen processing and presentation by antigen-expressing cells (12), but also for instance, as result of macrophage or dendritic cell-mediated phagocytosis upon innate checkpoint inhibition or chimeric antigen receptor (CAR)-mediated activity, only reported using transgenic primary T cells to date (55, 56).

In conclusion, the method presented here based on the E7_11-20_-specific TCR enabled rapid and efficient study of antigen-specific T cell responses *in vitro*, overcoming some of the main drawbacks of traditional transgenic primary T cells, including a shorter time of its protocol and the reduced cost of an immortalized reporter system. Overall, this luminescence-based approach can be used as a standardized tool to evaluate the impact of the immunosuppressive tumor microenvironment to optimize and speed up the development of novel immunotherapeutic strategies.

## Data availability statement

The original contributions presented in the study are included in the article/**Supplementary Material**. Further inquiries can be directed to the corresponding author.

## Author contributions

Conceptualization: EB and JÁ. Methodology: JÁ, YQ, LJ and ML. Validation: JÁ and YQ. Formal analysis: JÁ and YQ. Resources: GH, HL and EB. Data curation: JÁ and YQ. Writing—original draft preparation: JÁ, YQ and EB. Writing—review and editing: JÁ, YQ and EB. Supervision: EB. Project administration: EB. Funding acquisition: EB. All authors have read and agreed to the published version of the manuscript.
