# Supplementary material for: A luminescence-based method to assess antigen presentation and antigen-specific T cell responses for in vitro screening of immunomodulatory checkpoints and therapeutics
Source: Front Immunol. 2023 Jul 25;14:1233113. doi: 10.3389/fimmu.2023.1233113 (PMC10407562; doi:10.3389/fimmu.2023.1233113)
Supplement: Supplementary file 1 [file DataSheet_1.docx]

Supplementary Material

**A luminescence- cell based method to assess antigen presentation and antigen-specific T cell responses for *in vitro* study of immunomodulatory checkpoints and therapeutics**

**Jimena Alvarez Freile^1†^, Yuzhu Qi^1†^, Lisa Jacob^1^, Maria Franceskin Lobo, Harm Jan Lourens, Gerwin Huls, Edwin Bremer^1*^**

*** Correspondence:** Prof. Dr. Edwin Bremer e.bremer@umcg.nl


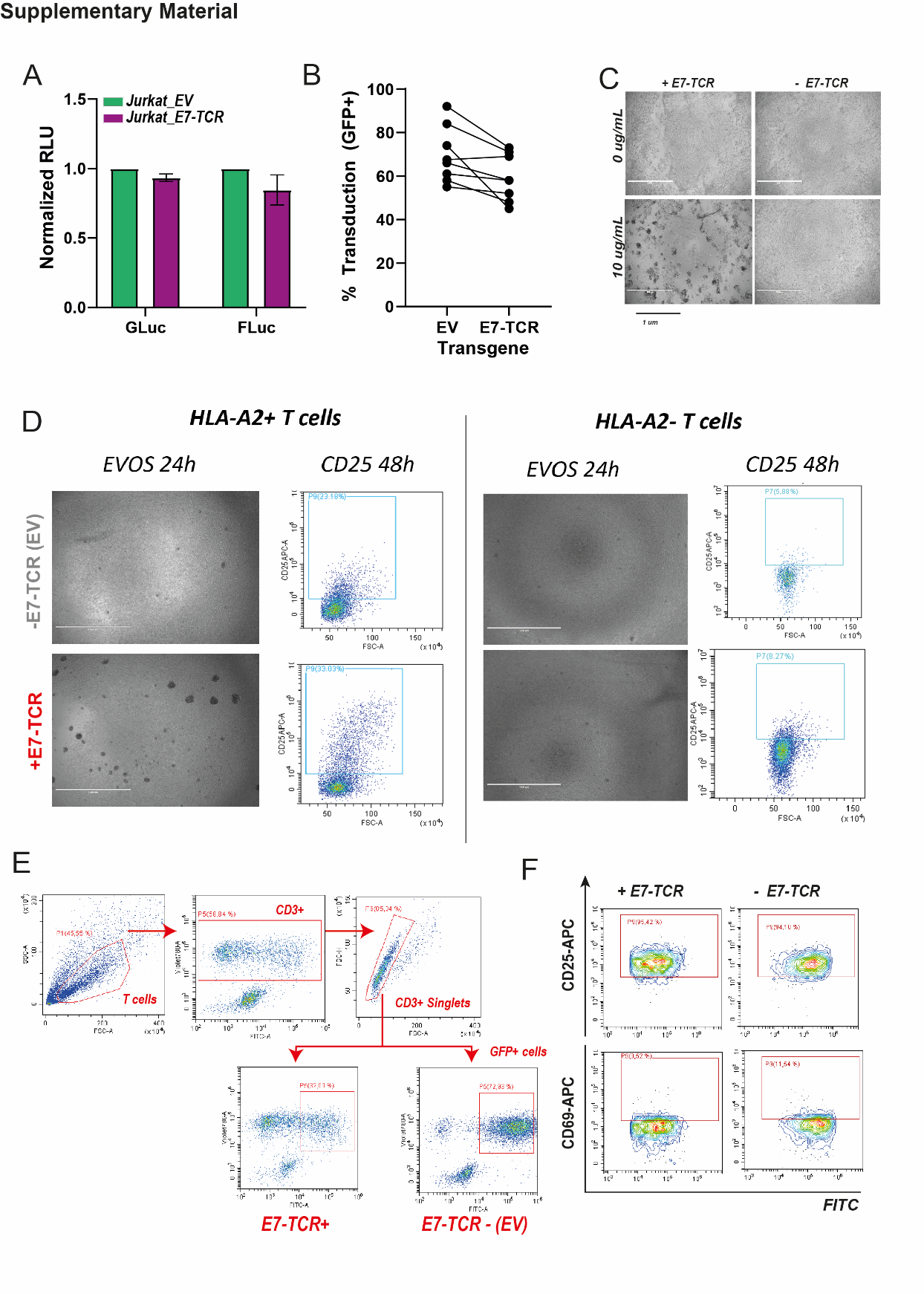


**Supplementary material part 1|** **(A)** Normalized induction of RLU by Jurkat.NFAT.Gluc and Jurkat.NFAT.Fluc expressing the E7-TCR transgene (purple) or not (green). Mean+SD n=3 **(B)** Percentage of E7-TCR transgene transduction among 8 different T cell batches measured as %GFP positive cells by flow cytometry. **(C)** Microscopy images (EVOS) after 24h co-culture between HEK 293T cells pulsed with 0 or 10 µg/mL of E7_11-20_ peptide with E7-TCR (+) or EV (- E7-TCR) transduced T cells. **(D)** Microscopy images (EVOS) and flow cytometry diagrams illustrating T cell clustering and T cell activation (CD25 upregulation) of HLA-A2^+^ and HLA-A2^–^ T cells expressing the E7-TCR transgene (red) or not (gray) and in the presence of 10 µg/mL of E7_11-20_. **(E )** Gating strategy scheme for FACS data analysis of CD25 and CD69 upregulation within CD3+ (BV785) E7-TCR/EV (FITC+) populations. **(F)** Flow cytometry diagrams for CD25 (APC) and CD69 (APC) upregulation within CD3^+^E7-TCR/EV cells upon 24h co-culture with HEK 293T cells without peptide.


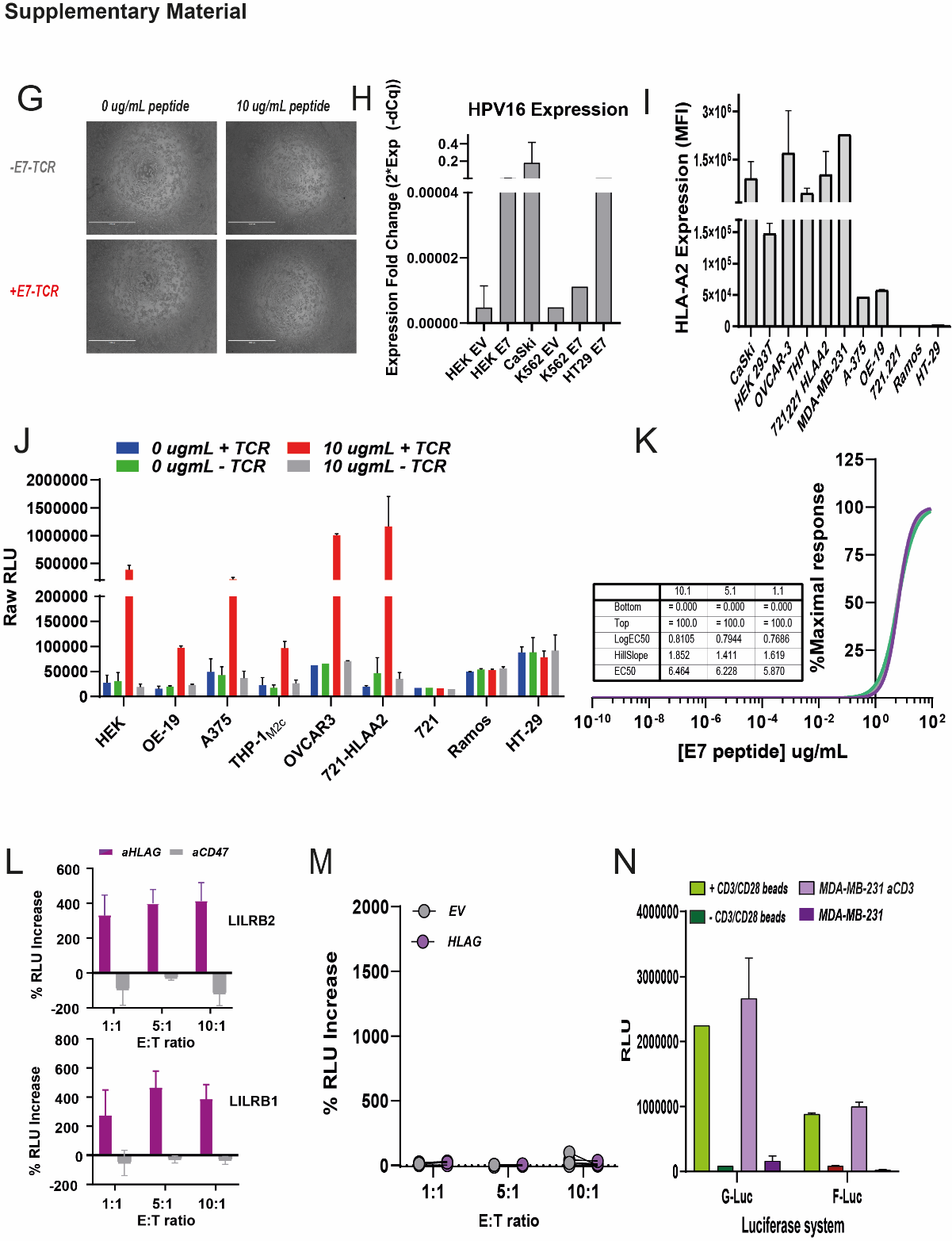


**Supplementary material part 2|** **(G)** Microscopy images (EVOS) of T cell clustering after 24h co-culture of T cells containing the E7-TCR transgene or not with HT-29 cells (HLA-A2^-^) in the presence of 0 and 10 µg/mL of E7 peptide. **(H)** mRNA levels of HPV16 among a panel of different cell lines obtained by RTqPCR. **(I)** HLA-A2 surface expression measured as MFI values of a panel of different cell lines **(J)** Absolute RLU values obtained for E7_11-20_-pulsed (10 µg/mL) and non-pulsed cells (0 µg/mL) HLA-A2^+^ (HEK 293T, OE-19, A-375, THP-1, OV-CAR-3, 721.221HLA-A2, MDA-MB-231) and HLA-A2^–^ (Ramos, HT-29, 721.221) cell lines upon 24h co-culture with E7-TCR (+TCR) or EV (-TCR) Jurkat^Fluc.E7-TCR^ cells. **(K)** Percentage of maximal response of Jurkat^Fluc.E7-TCR^ cells at different E7 peptide concentrations. **(L)** Percentage of RLU increase of Jurkat^Fluc.E7-TCR^ expressing LILRB1 (below) and LILRB2 (up) upon co-culture with E7-pulsed (10 µg/mL) 721.221.HLA-A2 cells expressing HLA-G and previously incubated with 5 µg/mL of anti-HLA-G or anti-CD47 antibodies. %RLU increase is calculated compared to non-pulsed cells. **(M)** (Percentage of RLU increase of Jurkat^Fluc.EV^ upon co-culture with 721.221HLA-A2.EV (gray) or 721.221HLA-A2.HLAG (purple) cells pulsed with 10 µg/mL of E7_11-20_ **(N)** RLU values generated by Jurkat^NFAT.E7-TCR^ Gluc and Fluc upon T cell activation with CD3/CD28 Dynabeads (green) or co-culture with MDA-MB-231 cells expressing or lacking the aCD3-scFv system (purple).


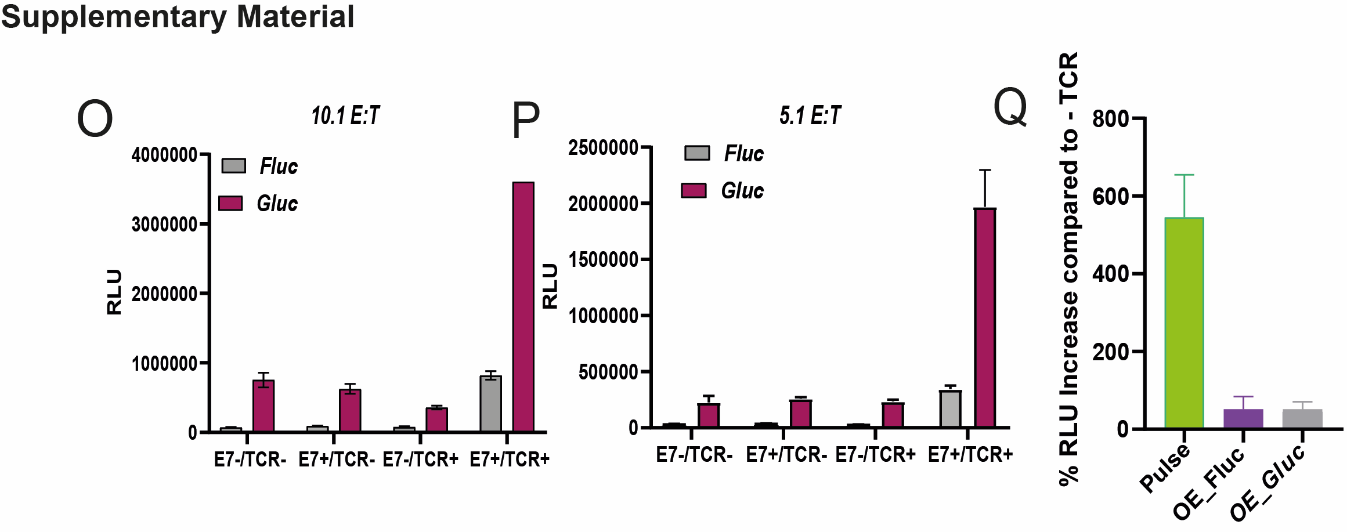


**Supplementary material part 3|** **(O)** RLU values generated by Jurkat.NFAT.E7-TCR (TCR+) or EV (TCR-) Gluc (purple) and FLuc (gray) upon co-culture with MDA-MB-231 (HLA-A2^+^) cells pulsed with o (E7-) or 10 µg/mL (E7+) of E7_11-20_ peptide. Cells were co-cultured at 10:1 E:T. **(P)** RLU values generated by Jurkat.NFAT.E7-TCR (TCR+) or EV (TCR-) Gluc (purple) and Fluc (gray) upon co-culture with MDA-MB-231 (HLA-A2^+^) cells pulsed with o (E7-) or 10 µg/mL (E7+) of E7_11-20_ peptide. Cells were co-cultured at 5:1 E:T **(Q)** Comparison between the % of RLU increase by Jurkat.Gluc.E7-TCR cells upon co-culture with polarized THP-1 cells previously pulsed with 10 µg/mL of E7_11-20_ peptide (defined as Pulse, green, n=5), expressing HPV16 (defined as HPV16 OE_Gluc purple, n=4) and same but instead of Gluc, the Jurkat.Fluc.E7-TCR system (defined as OE_Fluc).
